# Personal Views of the Management of Typhoid Fever

**Published:** 1905-07

**Authors:** Edward C. Register

**Affiliations:** Charlotte, N. C.; President of the North Carolina Medical Society; President of the Councillors of the Medical Society of North Carolina; ex-President of the Board of Medical Examiners of North Carolina; ex-President of the Charlotte Medical Society; Editor *The Charlotte Medical Journal*


### PERSONAL VIEWS ON THE MANAGEMENT OF TYPHOID FEVER.*

BY EDWARD C. REGISTER, M.D.,

CHARLOTTE, N. C.

President of the North Carolina Medical Society; President of the Councillors of the Medical Society of North Carolina; ex-President of the Board of Medical Examiners of North Carolina; ex-President of the Charlotte Medical Society; Editor *The Charlotte Medical Journal.*

While we are still far from any specific method capable of destroying the exciting causes of typhoid fever, or preventing its dissemination, or attenuating the activity of its toxins, we have perfected methods of treatment that are rational and scientific. In considering the treatment of this fever it is well enough to devote at least a paragraph to the subject of the general management of cases. We deal with no disease where carefully selected directions, properly carried into effect, bring us greater or more satisfactory returns. It is the little particulars more than even the intelligent use of drugs that deserve most consideration. Patients should be immediately placed in bed, certainly if typhoid fever is anticipated. By doing this we at once begin to curtail an unnecessary waste of vitality and physical force, that later on may play an important part in the saving of the life of our patient.

The removing of cases after they have contracted the disease is a question that often comes up, and it is not every time easy to make an intelligent or wise decision in certain cases.

We accept the general belief, and our experience confirms the idea, that it considerably increases the mortality in cases of this fever that have to be moved, certainly to any considerable distance. It usually necessitates the loss of a night's sleep, the omission of a day's medical attention, an improper diet for probably a day, with anxiety and worry and a loss of more or less physical strength. It is the best, as a rule, to advise patients to remain where they are, if the environments are suitable and if the disease has developed to a point where a correct diagnosis can be made. Prior to this,

*Read before the recent meeting of the North Carolina Medical Society at Greensboro.

my rule is to encourage patients to go where they can be best cared for. When the diagnosis has been made, and the surroundings are bad, even if the patient seems very sick, if other quarters are accessible and satisfactory and available, I often recommend the change.

It has always been a matter of doubt in my mind whether it is wise or not to invariably advise the use of the bedpan. I never in the majority of my cases insist upon its use. This practice is not exactly orthodox, and may cause adverse criticism. In most cases it is only necessary to consider but one thing when this question arises—it is this, will its use cause less physical exertion, less effort and less expulsive force and straining, than if the patient is carefully taken up in a semi-recumbent position. I hardly think so. There is no more straining on the bowels in a standing or sitting position than in the lying; there is a slight difference in the blood pressure, of course, in these different positions in health, and the same rule prevails in fever cases. It is a prevalent belief now that the straining and expulsive force and effort used to move the bowels in the lying position with the hips elevated, causes in the majority of cases fully as great blood-pressure and physical exertion as it will to let the patient be taken up. If the patient has a considerable diarrhea and is very weak with a tendency to hemorrhage, it might be well enough to encourage the use of the bedpan.

If practical the services of a competent nurse should be secured, members of the patient's own family are not, as a rule, satisfactory attendants. I prefer a trained nurse and a stranger. The termination of our cases will be greatly influenced by the skill and talent and faithfulness of our attendants. Sometimes competent and conscientious nurses make failures in certain cases. They do not seem to understand the peculiarities of the patient, or the patient does not appreciate the methods of his nurse, consequently discord prevails, just the condition that we want most to avoid. If the nurse is not satisfactory to the patient, if at all practicable, a change should be made. Nothing frets a case more than for him to think he is neglected, or improperly nursed.

The room of the typhoid case should be quiet, sunny, and well ventilated. It is always well enough to have two beds in the room, and let the patient lie on one during the day and the other at night. This may appear to be a small thing, yet there is a great deal more in it than most of us might think. It always makes a patient feel refreshed and better when he makes the change. He feels like he has gone to bed for the night, and is sure to rest better and feel more comfortable. The temperature of the room should range from 68 to 75 degrees if it is winter. Nurses should be carefully instructed about drafts. During the active fever stage there is very little risk in letting fresh air and cold air blow upon the patient, especially during the day if he is awake. If the temperature is running low and the patient is asleep, it is well enough to avoid drafts, but if he has any fever at all and is awake, a draft is almost without danger. If his temperature is normal and he is awake, avoid drafts. If his temperature is normal and he is asleep during the stage of convalescence, drafts are very dangerous. I have seen more than one life jeopardized on account of cold drafts during the latter stage of this disease. The doctor, however, in the country, or even in the city, who deals with newly-made nurses and attendants, can not think to warn them at all times about everything that may come up, consequently, in important fever cases, they should be competent to begin with. Nearly every physician believes that we should have a very light room for these patients. Experience has taught me that it is not always the best. Often a bright light causes headache, nervousness, and possibly delirium when a comparatively dark room is much more agreeable to the patient, and often enables him to sleep when a bright light would prevent it. A patient's choice about this is a much better guide for us to follow than an old rule that has been handed down to us for so many years. It may appear a trifling thing, yet I always consider it a good practice to inquire of all my fever patients how they like their beds. They should certainly be satisfied and pleased with them as much as it is possible. Sometimes they are too short, or too hard, too soft, or in ridges, and the physician and possibly the nurse will not know of it, and the patient will hardly have sense enough to complain. Looking after and preventing these little things that annoy and fret fever cases often saves a great deal of wear and tear and exhaustion of their nervous systems. It is often overlooked by nurses and even physicians, the importance of occasionally turning a delirious or weak patient over, from side to side and from back to side. I lost a patient once by neglecting to do this in the fourth week, on account of hypostatic congestion of the lungs. A mistake like this should never be made.

During the third week it is well enough to anticipate the presence of bed sores. A sudden rise of temperature about the twentieth day in this fever should always suggest this complication. They cause a fluctuating temperature in a larger per cent. of cases than is usually supposed. While the belief seems to prevail that the physician and nurse deserve reproach, when this dreaded sequela is in evidence, should be discouraged, we ought to recognize the fact that a suitable bed with proper attention given to cleanliness, bathing, etc., will reduce the occurrence of this complication to two or three per cent.

The mouth and teeth and nose of fever cases should be kept as clean as it is possible. It makes the patient much more comfortable. They should be washed several times a day with a solution of listerine and water. This possibly eliminates one of the sources of autoinfection, which very often produce, when all of the sources are included, just as much fever, just as much headache and restlessness and diarrhea, and I might add danger, as the typhoid poison itself.

In the general management of fever cases there is nothing that is of more importance than that of admitting visitors. They have caused more disastrous results and endangered the lives of more of my patients than any other one thing that I have had to contend with. Twenty years ago this opinion was not as well fixed in our minds as it is now, and I know of no other evidence that we are more proficient at the present time than formerly than this change in the care of fever cases. We have all observed the rise of temperature, the quick pulse, the restless wakeful nights that follow long conversations with friends who were kind but without tact.

A person's mind in typhoid fever should rest—it should be absolutely quiet. It is as important as the quiet and rest of the body. Anything that will cause the mind to act should be avoided. It is unwise to let fever cases in their convalescent stage, while they have any fever at all, to even look at pictures. This will make them think, and thinking is an action of the mind that ought to be avoided. To have any one read with fever is an objection well known. To have any one read to a patient is never allowed among my cases. For them to listen to a conversation, even if they do not participate in it themselves, is harmful. After the fever has entirely subsided, and after they have had no elevation of temperature for several evenings, I notice that patients very often want some company. It does not usually do them any harm at this stage, and I begin to let them receive their friends. During the active fever stage, members of the family should be excluded as much as it is possible. The importance of regulating the number of visitors in all kinds of fever cases now is so well known that we have little difficulty along this line, compared with a few years ago.

It is, indeed, a very difficult matter to properly nourish a typhoid case. When I can intelligently feed a fever case, I can usually give a favorable prognosis. We are fortunate if we can conduct a case through and at the end can not point to some period where we either overfed, or underfed, or fed improperly. It is well enough for us usually to adhere to some general rules. A patient should always be fed on fluid nourishment, and this should be kept up at least a week after the temperature has been normal. I am not in sympathy with the usual plan of giving nourishment at fixed intervals; this should be done as much as possible, but it is not always practicable to do this, especially if your patient is not a good sleeper. It is my custom to give nourishment every three hours while the patient is awake. It is seldom that I ever have a patient of this kind waked for the purpose of taking nourishment. We may sometimes find cases that sleep a great deal, and it might be necessary in these cases to disturb these long sleeps for the purpose of giving nourishment. I never have seen but one good sleeper, suffering from typhoid fever, die. I usually feel satisfied about a patient whenever fever produces sleep, and I always feel uneasy when fever produces insomnia. I consider it very unfortunate always when the patient will not sleep except when aided by medicines, and I am very slow usually to resort to medicines of this class. It is generally believed, and I am in sympathy with the belief, that milk is the best food for typhoid fever, and I usually prefer sour milk, or buttermilk. This may not be an ideal food, but it seems to be the best that I know of. It seems to pro-duce less nausea and less evidences of indigestion than anything else in my patients. It is less likely to produce tympanites, or diarrhea, than any other form of food. This may not be correct, but I believe that it is. I suppose that I give buttermilk as food in nearly eighty-five per cent. of my fever cases. I don't believe that I use sweet milk in over ten per cent. of these patients. This, apparently, ought to be the best food, especially if it is fresh and properly diluted with lime water and properly given. I very often add to it a little carbonated Harris Lithia Water. This makes it a very palatable food. It is my favorite in fever cases next to buttermilk. It is seldom that I give over half a glass of milk, either buttermilk or sweet milk, every three hours while the patient is awake, and my instructions usually are to keep this up during the night, if the patient is found to be perfectly awake, otherwise it is not given at all during the night, that is as a rule. I can, however, recall many cases in which I have made exceptions to this. If sweet milk is given it should be taken very slowly. I often suggest giving it with a spoon. This will prevent large solid curds in the stomach, and will make the milk easier digested. The milk diet, even if intelligently used, will not suit every case. It will cause gastric disturbance that may annoy the case no little, possibly produce nausea, vomiting, tympanites and diarrhea. These cases, in my hands, have been very few, not over five per cent. I can now recall but a few such cases. The milk may be replaced by soups, or animal broths; when this is necessary we often have to rotate from one to the other, as the patient tires on this food quicker than on milk. I have gotten in the habit lately of using Armour's Beef Extract, when a substitute for milk is necessary. This is easily prepared and very palatable. If there is any reason why this can not be given, I have prepared chicken tea, or beef tea. I make this preparation as follows:

Take one pound of tender chicken, or a pound of tender beef, beat it up thoroughly, put it in cold water enough to float it, gradually heat to a simmering point for over an hour, then boil for five minutes. The addition of salt and pepper make it a very delightful and nourishing food. It is a prevalent practice among some physicians, when sweet milk is not acceptable to the patient to add whiskey. This is a practice that I abandoned a great many years ago, never to take up again. Broth made from beef, mutton, veal, or chicken, with rice or barley or tomatoes, may be, after it is carefully strained, given to patients, and it is often relished and apparently suitable. But as I have intimated, I always feel a little uneasy when my patients have to be fed in this way. Peptenoids and panopeptone have their value, and they are usually accessible and easily prepared, and seem to be satisfactory in many cases. I have often fed cases through an entire attack of fever on one of these products alone. During the cold months we are asked about oysters and oyster soup. Oyster soup is as safe as any of the soups I have mentioned, and often very well received by the patient. I have known competent physicians to give raw oysters to fever cases. I may be wrong, but I admit that I have a great prejudice against this method. I am also greatly prejudiced against raw eggs given, as they usually are in these cases, beaten up in a glass of milk, often with whiskey and sugar added. Gruel, or any of the semi-solid foods, or milk shakes are equally as objectionable. Several years ago, with misgivings, I gave the white of eggs, and sometimes eggnog to typhoid cases, but soon abandoned the practice. I believe any one who can observe intelligently, and will observe, can easily see the evident mischief that this class of food often produces. It is a practice with some, and I have indulged in it myself to some extent, to give food every hour or two, but I have abandoned this method. You are likely to overdo the thing when you do this, and if we make a mistake at all in feeding a case, it is better to underfeed than to overfeed. A half glass of broth or milk or soup too much may cause a disorder that it will take days to correct.

Water should always be given freely. It will eliminate better than anything else the waste products that accumulate in the system. It should always be given at stated intervals if the patient is not thirsty, but as a rule he should never be disturbed when asleep. If he does not care to take it freely, he ought to be encouraged to do so. Usually I have a glass of ice water on a table by the bed, so the patient can take it ad libitum. If he neglects to take it himself, or is delirious, I have the nurse at least once every hour to give him a drink, and always note the amount taken in twenty-four hours. Usually a man weighing 150 pounds, during the active fever period, can easily take fifteen or twenty glasses in a day and night without any feeling of discomfort.

I often give lemonade; really I suggest it in nearly every case. I give the pure lemon juice and water. I seldom add sugar, as sweet lemonade always causes flatulence, and I have known it to produce nausea, vomiting and diarrhea. The juice of a lemon in a half glass of water, three or four times a day, is very refreshing, and is considered acid enough in fever cases.

Alcohol has a very limited use in the treatment of typhoid fever. Occasionally I give it, but always have a specific reason for doing so. I never have believed that it is in any sense a very valuable heart stimulant, and I never use it for this improbable action. It is not a heart stimulant proper, but may be so indirectly, like morphine, or any drug that has a tendency to quiet the nervous system. As a food it is of very little value. I have seen cases that were very restless and nervous and wakeful that whiskey would quiet and soothe when nothing else seems to do well. I have also seen it quiet delirious patients when other means failed. These cases are very few, for as a rule, it causes more nervousness, more irritability, more insomnia and more delirium. If it produces sleep and quiet and moisture of the tongue, it is indicated. Usually it makes the tongue drier; tends to wakefulness, restlessness and delirium. The number of cases of typhoid fever in my practice, where alcohol is indicated, will not exceed ten per cent. In severe hemorrhages, or in perforation, or in fact anything that causes a sudden depression of an already enfeebled condition, alcohol might be given to advantage. When the nervous system has become exhausted, and muscular weakness has come on gradually, and is well marked, alcohol is not of very much value. I always give fever patients who have passed the age of forty-five more whiskey than younger subjects, and if I find that the patient has been in the habit of taking a great deal of whiskey regularly, prior to his attack of fever, it is never withdrawn abruptly, but continued as the case indicates. It certainly, in such cases, requires more than in a person who is young and who has had temperate habits. In people past middle life, stimulants, especially strychnine and whiskey, are of great value. They are especially so with the aged, but we see typhoid fever very little in this class of patients. A great deal of injury is often done in giving young people alcohol in the active fever stage. Among my cases it has almost invariably produced more weakness and more irritability and more raving and loss of sleep than I believe would have existed without it. When I give it at all, I give few doses and large quantities. I have seen cases where fright and worry and anxiety were about to jeopardize my patient's life. In these cases nothing seemed to act better than a good drink of whiskey occasionally. It does away with that feeling of disquiet, and that feeling of fear and danger that often saps a great deal of the vitality of our patients. It is in this class of cases that we are often deceived about its real value as a heart stimulant. We find here a fast, irregular and weak pulse; a good dose of alcohol will make them slow and regular and full. Morphine would have a similar effect, but is not as desirable for reasons well known. I often give champagne for nausea that occurs during any stage of the disease.

Cold water is the best means of reducing high temperature. This is now generally admitted by all physicians. We never resort regularly to the tub bath; it is only in a small per cent. of my cases that we have used it. It is well enough to only use the tub bath when the cold packs and cold spongings are insufficient. Ninety-five per cent. of my cases are treated by cold spongings and cold packs, and usually I have very little difficulty with these means in keeping the temperature in the neighborhood of what is satisfactory to me. I use the cold pack in about thirty per cent. of my cases. My method of using it is as follows: The bed is protected with an oil sheet, on that is placed a woolen blanket, on that a wet sheet of the temperature of 60 to 65 deg. Fahrenheit; the patient is put on that and it is neatly and closely folded round him, then the blanket is fixed the same way, with a cold towel round the patient's head. This process is repeated about every fifteen or twenty minutes until the temperature is at a satisfactory point. As I say, this method is resorted to in not more than thirty per cent. of my cases. I never treat a case of fever without using the cold sponging, and it is not always either that I advise ice-cold water for this purpose. Sometimes the water has a temperature of 90 degrees, and sometimes, of course, as low as 60. It is a favorite plan of mine to add alcohol or vinegar or ordinary common salt to the water used in this sponging. Rubber bags filled with ice applied on the head or abdomen sometimes is a method of abstracting heat from the body, that I use. It is not sufficient in severe cases. There is no doubt that they are capable, if they are properly used, of taking from the patient a great deal of heat that he otherwise would have. The nurse is always instructed to remove the ice bag when the patient drops off to sleep; especially is this precaution necessary in cases where the fever is on the decline and is apt to approach the normal. In such cases it would be sure to produce a subnormal temperature and possibly bronchitis, neuralgia, or some lung complication that might cause us anxiety and possibly endanger the patient's life.

The medicinal treatment of the disease has been greatly depreciated in the last few years, more so than it should be. Few of us conduct a case through without prescribing several different drugs, with no idea of aborting or curing it, but to modify symptoms and prevent complications. We will take up systematically the drugs used here. One of the oldest and still one of the prominent drugs used in typhoid fever is quinine. It has been used in this disease ever since the two have been known, and to-day in the hands of some it is a popular antipyretic. It has been my practice in the beginning of all fevers to give quinine in large doses when the fever intermits; that is when the patient has fever only in the afternoon, and it reaches normal in the early morning. With the proper diet and thorough elimination and rest, with large doses of quinine, if the fever continues for five or six days, I conclude in all probability that it is a typhoid attack, unless it is due to some local cause that is easily recognized. In the latter part of an attack of typhoid fever, if the patient is not very much exhausted and the fever reaches normal in the morning and runs up a degree and a half to two degrees in the afternoon, quinine will often shorten these afternoon fevers, but whether it favorably influences the pathological lesions that produce these afternoon fevers, is questionable, and whether you really hasten a satisfactory convalescence is still doubtful in my mind. I do not invariably follow this plan now, but at one time, in certain cases, it was almost my routine practice. I always give it in large doses at night; it acts better then. It never does any harm in any case, even in large doses, if it produces sleep, but I am rather doubtful at all times of the wisdom of its use, if it produces insomnia, I mean in typhoid fever. Antipyrine, phenacetine and antefebrin have all been used to reduce fever. Some of them are used now, but to no great extent. It is extremely uncommon that I prescribe any of these drugs at all in this fever. They are invaluable in fevers of short duration, with the exception of pneumonia. Certainly all fevers of long duration do not call for this class of drugs.

There is unquestionably a great deal in the antiseptic treatment of the disease. It does not in any way, so far as I have been able to see, cut short its duration, or have any tendency whatever to abort or cure an attack, but it will relieve or modify very important pathologic conditions that cause ugly symptoms. A great many antiseptic drugs have been recommended, but I am inclined to believe that there is not, among the best ones, so very much difference in the effect. I have used calomel, carbolic acid, tincture of iodine, turpentine and salol more than any other drugs. In the beginning of a continued fever, if I anticipate typhoid, I give good doses of calomel, enough to bring about a decided physiological effect. After the first few days it has not been my custom to use it, although I have used the drug in all stages of the disease. When constipation is present, I have prescribed one-tenth of a grain of calomel every hour, or every two hours, or every three hours until the bowels act. This has not been my custom in the last few years. I usually recommend the use of enemas. I would not, however, criticise any doctor who would have a desire to follow this plan that I have practically abandoned. The carbolic acid and iodine that were so popular fifteen years ago have not been used in my practice for several years. When I use a drug at all with the hope of getting an antiseptic action of the bowels, I use salol. As I have said, it may not be any better than the others, which have equally as good reputation, yet it is the drug that I use. It brings about the results that I give it for, namely: it flattens the bowels when tympany exists; it checks diarrhea; it brings down the temperature, and makes the evacuations almost odorless; it causes very little disturbance of the stomach, and its administration carries with it as little danger as that of any drug. I usually give it in powders, and I have not modified my plan, so far as the quantity is concerned, for several years. When I select salol as a bowel antiseptic, I give it in much larger doses than that usually suggested in most of the text-books. My maximum quantity to be given in the twenty-four hours was formerly about sixty grains. This quantity is in most cases sufficient, but in nearly half of my cases where the temperature has a tendency to run very high, and where there is a considerable amount of tympany and a decided tendency to diarrhea, I often give as much as fifteen grains every three hours, and in a few cases I have given as high as twenty grains every three hours. There is plenty of authority now to justify this practice, but there was not when I first adopted it. I can recall very well that I have often instructed nurses not to put on their charts the quantity of salol given, for fear that my plan might be considered too radical. I don't know that I have ever given over one hundred and twenty grains in a day and night, but as I have said, I reach this amount occasionally. In doses of this kind it is a decided antipyretic. It should never be given in this quantity unless the conditions that I have mentioned exist classically. Recent experiments have established facts that have been a source of gratification to me, and have strengthened my belief that salol is a good disinfectant for the intestine, possibly the best.

Retained urine is a common thing in typhoid fever, and it is commonly known among physicians now that it does in a great majority of cases contain many typhoid bacilli, and that they are often reabsorbed, and that this is one of the sources of reinfection, or autoinfection. I have never demonstrated it, but it has been shown by apparently reputable observers that forty grains of salol in twenty-four hours will perceptibly lessen the number of the typhoid bacilli in the urine, and that eighty grains per day will almost eliminate them entirely.

I am greatly prejudiced against what is usually known as the eliminative treatment in typhoid fever. I mean alimentary elimination. I have already mentioned my ideas of the elimination in the treatment of fever cases that is necessary. If the bowels are thoroughly washed out during the first few days of the attack, I seldom, if ever, recommend the further use of alimentary eliminatives. I know theoretically it is a plausible plan, and it looks like it would be well enough, when you give drugs to disinfect the contents of the alimentary tract, that it would then be wise to wash it away; that is, the exudate, the sero-purulent fluid from the ulcerated surface of the bowels. This is a beautiful theory, but in actual practice it will not do. I hope I will not be considered discourteous or dogmatic to depreciate in any way the opinion of others, but I am so convinced that purgatives—I mean active purgatives—during the second and third week of typhoid fever, is so commonly the cause of mischief in my cases, that I am actually afraid of the procedure. I never propose again to take, or share the consequences or responsibilities in any case where this practice is insisted upon by my associate. My cases have never done well with such treatment. It is certainly in my opinion not a rational procedure.

The management of certain symptoms and complications, such as headache, insomnia, delirium, vomiting, diarrhea, constipation and hemorrhage deserve our consideration. I will pass over these headings briefly. For headaches, unless it is a very severe form, medicines are not indicated. A cold ice bag to the head, and sometimes hot applications, will relieve it. It usually passes off after the first week. It is always due to improper elimination, or indigestion, or assimilation, or diet. As soon as these functions are better established, the headache passes away. In only a very small per cent. of cases does it continue after these corrections have been made. When it does, I am in the habit of giving some simple remedy, like tablets of antikamnia and codeine; a few of these are usually sufficient to relieve the most obstinate cases. Insomnia in typhoid cases always gives me great concern and anxiety. These cases, so far as I have observed, never do well; a large per cent. of them die. Hypnotics here, as a rule, are unsatisfactory; their action is very uncertain. Opiates, one of our most reliable hypnotics, will only produce sleep about one time in ten in my cases. Chloral, about four times out of five. Trional and sulphonal about one time in three. Whiskey about one time in twenty. After the best of these act beautifully, I never detect the refreshing effects from them that I anticipated. When I leave my patient tossing about all night in a semi-delirious state, getting a little nap here and there, making a total of some two or three hours, I find that his condition is more satisfactory in the morning than if I had produced an artificial sleep of seven or eight hours. This hardly seems plausible or rational in such cases, but such has been my experience. It is seldom that I use hypnotics in typhoid fever. In delirium I have never found any drug that is very satisfactory. Possibly bromide of potassium is the most satisfactory of them, although I have used valerian, dover's powders, whiskey, and hyoscine. The hyoscine did not come up to what I expected. In about five per cent. of my cases large doses of whiskey act very well; in the remaining cases it seems to aggravate the condition. Hot sponge baths, with a great deal of friction, sometimes has a very soothing effect. In vomiting usually one drop of carbolic acid in a teaspoonful of lime water and cracked ice will act satisfactorily in a great majority of cases, in connection with a mustard poultice on the stomach and the suspension of diet and drinking for a few hours. If this is not sufficient when properly employed, a glass of water at an ordinary temperature should be given. Sometimes this sticks and relieves the intense thirst that very often accompanies these attacks of nausea. I have often used a hypodermic of morphine in such cases. The remedy, however, that will control the largest number of cases is champagne in a little crushed ice. Cocaine and bismuth in a great many cases acts beautifully.

Tympanites is usually due to two distinct causes; one is amenable, the other is not. When the swelling is due to fermentation of products that lie in the alimentary canal it is of trifling consequence, and can usually be remedied by antiseptic measures and a slight laxative and correction in the diet. But if it is due to paresis, that comes on in the latter stage of very severe cases, it is entirely a different proposition. This means almost sure death. When we feel confident and have every reason to believe that we have fed our case intelligently, and that there is nothing remaining in the alimentary canal that would produce distention by fermentation, and our patient shows all signs of weakness, like delirium, weak pulse, and sagging of the eyelids, with extreme tympanites, we have a condition to deal with that is very dangerous and one that can not be relieved. When the tympanites is due to paresis, the use of turpentine and large doses of strychnine are about the only things that can be done. Enemas and purgatives and laxatives seem, in these cases, to aggravate this condition rather than relieve it. The use of the long rectal tube has been used in many of my cases, but with no very great benefit. I have seen a few cases that were restless with a considerable delirium and evidence of pain that were brought about and continued for days at a time by the retention of urine that had been overlooked. This is a mistake that ought never to be made. It is a usual practice of mine, if I have a case of fever that is extremely ill, and especially if they are delirious, to run my hand over the region of the bladder and examine especially for this common complication. I can recall many cases where this condition was overlooked.

A persistent diarrhea, one that can not be controlled, always alarms me. The old idea that two or three or four stools in the twenty-four hours were beneficial and should be encouraged, is entirely contrary to my idea of the subject. The mortality of my cases that have had this amount of diarrhea for ten days at a time has amounted to fully twenty per cent. On the other hand I am yet to see a case of typhoid fever die that was constipated. When by ordinary means we can keep the bowels from acting for three or four days at a time, the mortality in this part of the country will not reach five per cent. This has been my experience. After the first week to encourage what is supposed to be a harmless diarrhea and the persistent use of alimentary eliminatives are methods that I believe are dangerous. To encourage peristalsis when the bowel is congested and inflamed and ulcerated is, as it appears to me, very irrational. The retained secretions of the alimentary canal that concerns, really alarms, so many physicians if let alone does very little harm. This canal is in the habit of taking care of this kind of material. When it is disturbed the poison must multiply and absorption must take place, for our patient usually has a quicker pulse, a chilly feeling, more fever, more tympanites, more restlessness and more delirium.

I usually give bismuth and opium combined with salol for these cases of diarrhea. I never let the bowels remain unmoved for ten days to two weeks at a time, as some do, although I have seen many cases of this kind, and have never yet noticed anything to regret about the practice. I encourage the bowels to act every four or five days during the second and third and fourth week. To do this I recommend enemas of water; sometimes I add a little glycerine or turpentine.

When I meet with cases of hemorrhage, I am always alarmed. It is a much more serious complication than the text-books lead us to believe. Fifty per cent. of my cases have died with a history of hemorrhage. It has been suggested when hemorrhage occurs during the first week, that it is not a bad sign. I have never had a case where it occurred during that period. Treatment in such cases is very simple. I give opiates freely, keep the patient absolutely quiet, an ice-bag to the bowels, and almost a suspension of food for a day or two. The opiates should always be given in a liquid or powdered form, and in fact all medicines in a severe case should be given this way. I have observed at least a dozen times evidences that the alimentary juices were unable to dissolve capsules or pills. Some few years ago I drifted into the habit, in cases of diarrhea or hemorrhage, of giving the prepared pill of opium and nitrate of silver, and in a large per cent. of my cases I noticed no benefit whatever. When I would change to some form of opiate that would certainly be absorbed, my patient would show the physiological effects of the opium immediately. I have never been able to observe any benefit from the administration of ergot, although it is usually given in my cases. Astringents given by the mouth can not possibly reach the seat of the disease for many hours, and even whenever they do, their action may be doubtful. I usually encourage the plan, however, of giving silver or lead in such cases. In cases like this, where a tendency to collapse is present, the use of whiskey can be given with much benefit.

In the management of convalescent fever cases we have again to contend with the problem of how to feed our patient. When the temperature has remained normal in the afternoon for a week I give one reception cracker in half a glass of milk every three hours. If this is satisfactory and produces no harm, the next day I give two crackers in a half glass of milk every three hours. If my patient continues hungry and does not tire of this diet they are increased every day. The fourth day I give a soft-boiled egg with toast three times a day in connection with the crackers and milk, and gradually increase the diet in this way. A patient can eat twice as much, without any evidence of indigestion or being overfed, by remaining in bed; consequently I keep my patients there for two weeks after I begin to feed them, then there is but very little cause for uneasiness about mistakes in diet after the patient gets up. I have often seen cases at the end of an attack when the fever was normal in the morning, but slightly elevated in the afternoon, fret and worry so much about eating that it would in all probability be responsible for this degree, or degree and a half of fever. In such cases it is well enough to give a few crackers and possibly a soft-boiled egg and note the result. If it satisfies the patient, the fever is from a pathologic cause and not of nervous origin.

---

It is said that food laboratories will be established in Boston, New Orleans, and San Francisco similar to the one recently opened in New York City.

